# Dietary corn resistant starch regulates intestinal morphology and barrier functions by activating the Notch signaling pathway of broilers

**DOI:** 10.5713/ajas.19.0967

**Published:** 2020-03-12

**Authors:** Yingying Zhang, Yingsen Liu, Jiaolong Li, Tong Xing, Yun Jiang, Lin Zhang, Feng Gao

**Affiliations:** 1 College of Animal Science and Technology, Key Laboratory of Animal Origin Food Production and Safety Guarantee of Jiangsu Province, Jiangsu Collaborative Innovation Center of Meat Production and Processing, Quality and Safety Control, Joint International Research Laboratory of Animal Health and Food Safety, National Experimental Teaching Demonstration Center of Animal Science, Nanjing Agricultural University, Nanjing 210095, China; 2 School of Food Science and Pharmaceutical Engineering, Nanjing Normal University, Nanjing 210097, China

**Keywords:** Corn Resistant Starch, Intestinal Morphology, Barrier Function, Broiler

## Abstract

**Objective:**

This study was conducted to investigate the effects of dietary corn resistant starch (RS) on the intestinal morphology and barrier functions of broilers.

**Methods:**

A total of 320 one-day-old broilers were randomly allocated to 5 dietary treatments: one normal corn–soybean (NC) diet, one corn–soybean–based diet supplementation with 20% corn starch (CS), and 3 corn–soybean–based diets supplementation with 4%, 8%, and 12% corn resistant starch (RS) (identified as 4% RS, 8% RS, and 12% RS, respectively). Each group had eight replicates with eight broilers per replicate. After 21 days feeding, one bird with a body weight (BW) close to the average BW of their replicate was selected and slaughtered. The samples of duodenum, jejunum, ileum, caecum digesta, and blood were collected.

**Results:**

Birds fed 4% RS, 8% RS and 12% RS diets showed lower feed intake, BW gain, jejunal villus height (VH), duodenal crypt depth (CD), jejunal VH/CD ratio, duodenal goblet cell density as well as *mucin1* mRNA expressions compared to the NC group, but showed higher concentrations of cecal acetic acid and butyric acid, percentage of jejunal proliferating cell nuclear antigen-positive cells and delta like canonical Notch ligand 4 (*Dll4*), and hes family bHLH transcription factor 1 mRNA expressions. However, there were no differences on the plasma diamine oxidase activity and D-lactic acid concentration among all groups.

**Conclusion:**

These findings suggested that RS could suppress intestinal morphology and barrier functions by activating Notch pathway and inhibiting the development of goblet cells, resulting in decreased mucins and tight junction mRNA expression.

## INTRODUCTION

The small intestine is not only responsible for digestion and absorption of nutrients, but also plays critical roles in mucosal barrier functions, signal recognition and production of some endogenous biological active molecules [1–3]. Intact mucosal development and barrier functions are essential for intestinal health. Diet is one of the important factors shaping intestinal mucosal morphology and barrier functions. Starch serves as a major source of carbohydrates in poultry diets, which can be classified into rapidly digestible starch, slowly digestible starch and resistant starch (RS) according to its digestion and absorption rates in small intestine [4]. RS, which acts as dietary fibre, could resist digestion in small intestine and thus reach the large intestine where short chain fatty acids are produced by gut microbiota [5]. Therefore, RS exerts a prebiotic influence on mammalian intestinal health [6,7].

Complete mucosal morphology is the premise of normal intestinal digestion and absorption. Some animal studies have shown that dietary RS supplementation can increase the villus height (VH) [8], decrease crypt depth (CD), and promote VH/CD ratio [9]. It’s well known that the continuous renewal of intestinal epithelium is the result of the proliferation of crypt cells and their transfer to the top of villi, and then these crypt cells gradually differentiate into cells with various physiological functions such as absorption or secretion in the process of transfer [10]. Notch is a major regulatory pathway that determines the fate of cell differentiation of cryptic stem cells [2,11,12]. There are four core elements in the Notch signaling system, Notch receptor, Delta/Serrate/Lag2 (DSL) ligands, CBF-1, Su(H), Lag-1 (CSL) transcriptional cofactors, and target genes such as the hairy/enhancer of split (HES) genes family [13]. Notch activity affects the implementation of differentiation, proliferation, and apoptotic programs [14]. Generally, Notch inhibition leads to precocious differentiation of epithelial progenitors into secretory cell types, including large numbers of cells that expressed both Paneth and goblet cell markers [12].

There are four main cell types in intestinal epitheliums, including absorptive enterocytes, goblet cells, Paneth cells, and enteroendocrine cells [15]. among which goblet cells play a critical role in maintaining the intestinal barrier function via synthesizing and secreting mucin glycoproteins (MUC1, MUC2, MUC3, MUC17) [16,17]. The higher proliferation rate of intestinal epithelium is associated with lower differentiation and maturation, which may affect the physiological functions of the small intestine [18,19]. Different from mammals, birds have a shorter digestive tract and weaker microbial fermentation [20]. Although some achievements have been obtained in the studies on humans and mammals, little is known on how dietary RS affects intestinal morphology and barrier functions of poultry. Therefore, this study aimed to investigate the effects of dietary grade levels of corn RS on growth performance, intestinal morphology and barrier functions, as well as the changes of Notch pathway in intestinal mucosa of broilers.

## MATERIALS AND METHODS

### Ethics statements

All animal work was carried out according to the approved guidelines established by the Ministry of Agriculture of China. All experimental design and procedures were approved by the Institutional Animal Care and Use Committee of Nanjing Agricultural University (GB14925, NJAU-CAST-2011-093).

### Animals and experimental design

A total of 320 newly hatched one-day-old Arbor Acres broiler chicks were purchased from a commercial hatchery (Hewei Agricultural Development Co. Ltd, Xuancheng, China). Chicks were weighed and randomly assigned to five dietary treatments: one normal corn–soybean (NC) diet, one corn–soybean–based diet supplementation with 20% corn starch (CS) (maize starch, 99% purity, YuXing Inc., Hebei, China), and 3 corn–soybean–based diets supplementation with 4%, 8%, and 12% corn RS (identified as 4% RS, 8% RS, and 12% RS, respectively) by replacing CS with 6.67%, 13.33%, and 20% of Hi-Maize 260 (type II RS, 60% purity; Ingredion Inc., Westchester, IL, USA), respectively. All diets were formulated to satisfy the nutritional requirements according to the National Research Council (NRC, 1994). The composition and nutrition levels of all diets are given in Table 1. Each treatment group had eight replicates (one replicate per cage) with eight broilers per replicate. The birds were given free access to feed and water in a temperature-controlled room at Nanjing Kangxin Poultry Industry (Nanjing, China) throughout a 21-d experiment. On 21 days, birds were weighed per replicate cage, and feed consumption was recorded by replicate to calculate the feed intake, body weight (BW) gain and feed/gain (F/G).

### Sample collection

At 21 days of age, one bird with a BW close to the average BW of each replicate was selected, weighed, stunned electrically and slaughtered immediately via exsanguination of the left carotid artery. Approximately 5 mL of blood samples were collected into heparinized tubes and centrifuged immediately to get plasma. After evisceration, the small intestine of birds was carefully separated into three segments: duodenum (from ventriculus to the pancreo-biliary duct), jejunum (from pancreo-biliary duct to yolk stalk), and ileum (from yolk stalk to ileocecal junction). The contents of each part was washed with pre-cold saline and weighted to calculate intestine index (relative weight = intestine weight, g/BW, kg; relative length = intestine length, cm/BW, kg). Approximately 1 cm of the middle portion of each intestinal segment were collected and fixed in 4% phosphate-buffered paraformaldehyde for morphological analysis. The mucosa of duodenal, jejunal and ileal was then gently scraped and digesta of the caecum from each bird were mixed and collected, frozen in liquid nitrogen and stored at −80°C for further analysis.

### Intestinal morphology

Intestinal samples were embedded in paraffin and cross sections (5 μm) were cut and stained with hematoxylin–eosin (HE) staining. Images were captured using an Olympus DP12 CCD digital camera (Olympus Optical Co. Ltd, Tokyo, Japan) and analyzed by Image-Pro Plus 6.0 software (Media Cybernetics, Bethesda, MD, USA) to measure the VH (μm, from the tip of villus to the villus-crypt junction level for 10 villi per section) and CD (μm, the vertical distance from the villus-crypt junction to the lower limit of the crypt for 10 corresponding crypts per section). The ratio of VH to CD (μm/μm) was also calculated.

### Goblet cell counts

Intestinal cross sections from the samples embedded in paraffin were stained with Alcian Blue and periodic acid–Schiff reagent (Sigma, St. Louis, MO, USA) for goblet cell counts. The microscopic image of each sample was examined at a final magnification of ×200. The density of goblet cell was defined as the goblet cell count per 100 μm villus length (n/per 100 μm villus). The average values of the density of goblet cells of 6 villus from duodenum, jejunum and ileum cross sections per chicken were used.

### Proliferating cell nuclear antigen immunohistochemistry

Immunohistochemical analysis was used to evaluate expression of proliferating cell nuclear antigen (PCNA)-positive cells. Cross sections (5 μm) of each bird were deparaffinized in xylene, dehydrated in a graded series of alcohol and rehydrated in phosphate buffer saline (PBS) (pH 7.4). Then, the sections were pre-treated by a microwave, treated with 3% hydrogen peroxide and incubated with primary antibody (Anti-PCNA mouse monoclonal antibody, 1:200 dilution; Bio Basic Canada Inc, Markham, ON, Canada) overnight at 4°C. After washing with PBS, the sections were incubated with the second antibody (Goat Anti-Mouse IgG for IHC/HRP; Bio Basic Canada Inc, Canada) for 45 min then washed again and treated with a diaminobenzidine staining kit (K5007; Angle Gene Bioengineering Co. Ltd., Nanjing, China), then finally counter-stained with hematoxylin. The analysis was carried out on histological slides at a final magnification of ×400. Approximately 200 cells in the middle portion of the well-oriented villus in the section were evaluated. The percentage of PCNA-positive cells for each villus was calculated as the number of PCNA-positive cells divided by the total number of cells counted, multiplied by 100. Values represent the mean of 6 villi from each bird.

### RNA extraction andreal-time quantitative polymerase chain reaction analysis

Total RNA was isolated from the duodenal and jejunal mucosa samples using Trizol reagent (Takara Biotechnology Co. Ltd., Dalian, China). The purity and quantity of the RNA were measured with a Nanodrop ND-1000 spectrophotometer (Thermo Scientific, Wilmington, DE, USA). Reverse transcription of total RNA was completed using a PrimeScript RT Master Mix kit (Takara Biotechnology, China). The RT products (cDNA) were stored at −20°C. Real-time quantitative polymerase chain reaction (RT-qPCR) was performed using the QuanStudio6 RT-qPCR detection system (Applied Biosystems, Foster City, CA, USA) using SYBR Premix Ex Taq kits (Takara Biotechnology, China). The following cycling conditions were used: 95°C for 30 s, followed by 40 cycles of denaturation at 95°C for 5 s and anneal at 60°C for 30 s, and the collection of the fluorescence signal at 60°C. All the specific primers used are listed in Table 2. The expression of target genes relative to 18S rRNA was calculated using the 2^−ΔΔCT^ method [21].

### Analysis of plasma diamine oxidase activity and D-lactic acid concentration

The diamine oxidase (DAO) activity and D-lactic acid concentration in plasma were determined with the commercial diagnostic kits (Catalog numbers: H263 and A088-1-1, respectively; Nanjing Jiancheng Bioengineering Institute, Nanjing, China) according to the manufacturer’s instructions.

### Short-chain fatty acids analyses

Approximately 0.3 g frozen cecal digesta was diluted with 5-fold double-distilled water in sterile Eppendorf tubes. The sample solutions were homogenized and centrifuged (15 min, 4,000×*g*). Supernatant (1.0 mL) was transferred to a new Eppendorf tube and mixed with ice-cold metaphosphoric acid solution (0.2 mL; 25% concentration; internal standard was added, crotonic acid 75.08 mmol/L). The solution was kept at −20°C overnight and then centrifuged (10 min, 16,000 ×*g*). Concentrations of the short-chain fatty acids (SCFAs) were determined in the supernatant using a Gas Chromatographic System (Agilent 7890A, Santa Clara, CA, USA).

### Statistical analysis

All data were analyzed using one-way analysis of variance using SPSS software (Version 20.0, SPSS Inc., Chicago, IL, USA). The effect of increasing concentration of dietary RS was determined by orthogonal polynomial contrasts. The model included linear and quadratic contrasts for effects of supplemental RS. The results are presented as means with their standard errors. A total of eight replicate were used per treatment (n = 8). Significant differences were declared when p<0.05.

## RESULTS

### Growth performance

As shown in Table 3, birds fed CS and RS diets had lower feed intake and BW gain than those fed the NC diet (p<0.05) and had higher F/G than those fed the NC diet (p<0.05). The feed intake increased linearly, and F/G increased linearly as the proportion of dietary RS increased (p<0.05).

### Development of small intestine

According to Table 4, birds fed CS and RS diets had higher relative lengths of small intestine (duodenum, jejunum and ileum) than those fed the NC diet (p<0.05). Birds from 8% RS and 12% RS groups and those from 8% RS, and 12% RS groups had higher relative weights of duodenum and jejunum, respectively (p<0.01). In addition, the relative lengths and weights of duodenum increased linearly as the proportion of dietary RS increased (p<0.05).

### Intestinal morphology

It is obvious in Table 5, the duodenal CD of birds fed RS diets was lower than those fed NC diet (p<0.01). Birds of CS group and RS groups exhibited lower jejunal VH and VH/CD compared with NC group (p<0.01). The ileal VH in groups of CS and 4% RS and the ileal CD in groups of 4% RS and 8% RS were lower than that of control (p<0.05). Besides, feeding RS diets linearly increased the ileal VH, jejunal and ileal CD, duodenal VH/CD, and linearly decreased the duodenal CD (p<0.01). Moreover, the jejunal VH/CD presented quadratic response to the increased RS in diets (p<0.05).

### Intestinal goblet cell density

**Figure 1:**
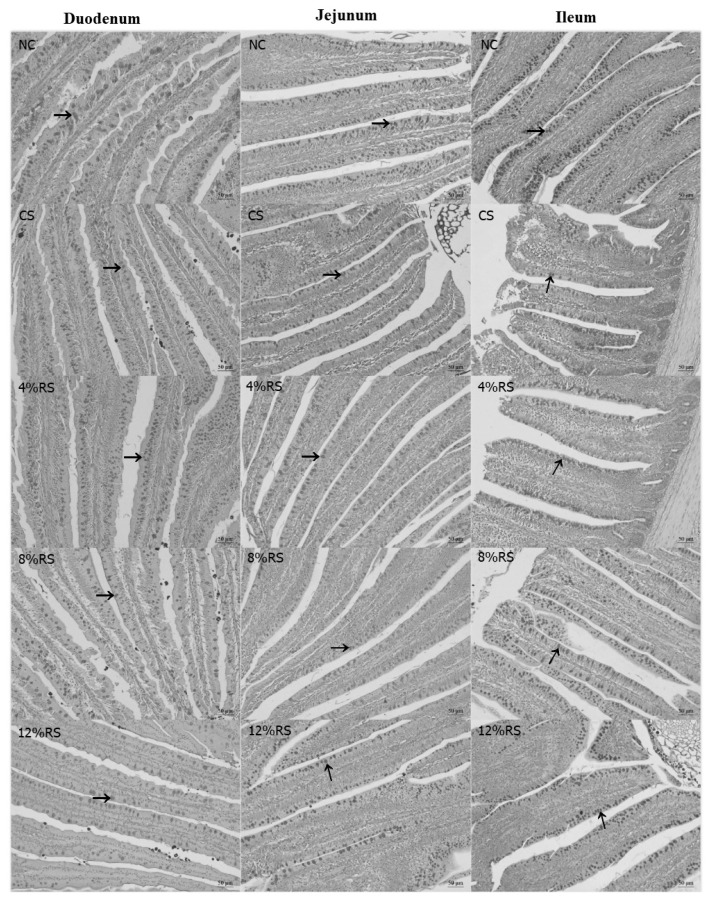
The periodic acid–Schiff staining of goblet cells in duodenum, jejunum and ileum villus of 21-day-old broilers (Arrows point to goblet cells). NC, a basic normal corn–soybeandiet; CS, a corn–soybean–based diet supplementation with 20% corn starch (CS); 4% RS, 8% RS, and 12% RS, the corn–soybean–based diets supplementation with 4%, 8%, and 12% corn resistant starch (RS), respectively. Image, magnification at ×200.

The Figure 1 shows that the duodenal goblet cell density (GCD) in 4% RS, 8% RS, and 12% RS groups were lower than that in NC group (p<0.05, Table 6), and the ileal GCD were decreased quadratically in response to the increase of dietary RS level (p<0.05).

### Effects on short-chain fatty acids concentration in cecal digesta

In comparison with the control group, higher concentrations of acetate and butyrate were found in the CS and all RS treatments, as well as a higher propionate concentration was present in birds treated with 8% RS and 12% RS diets (p<0.05, Table 7). Furthermore, propionate concentrations were increased linearly with the increase of dietary RS level (p<0.05).

### Immunohistochemical assessment of proliferating cell nuclear antigen

**Figure 2:**
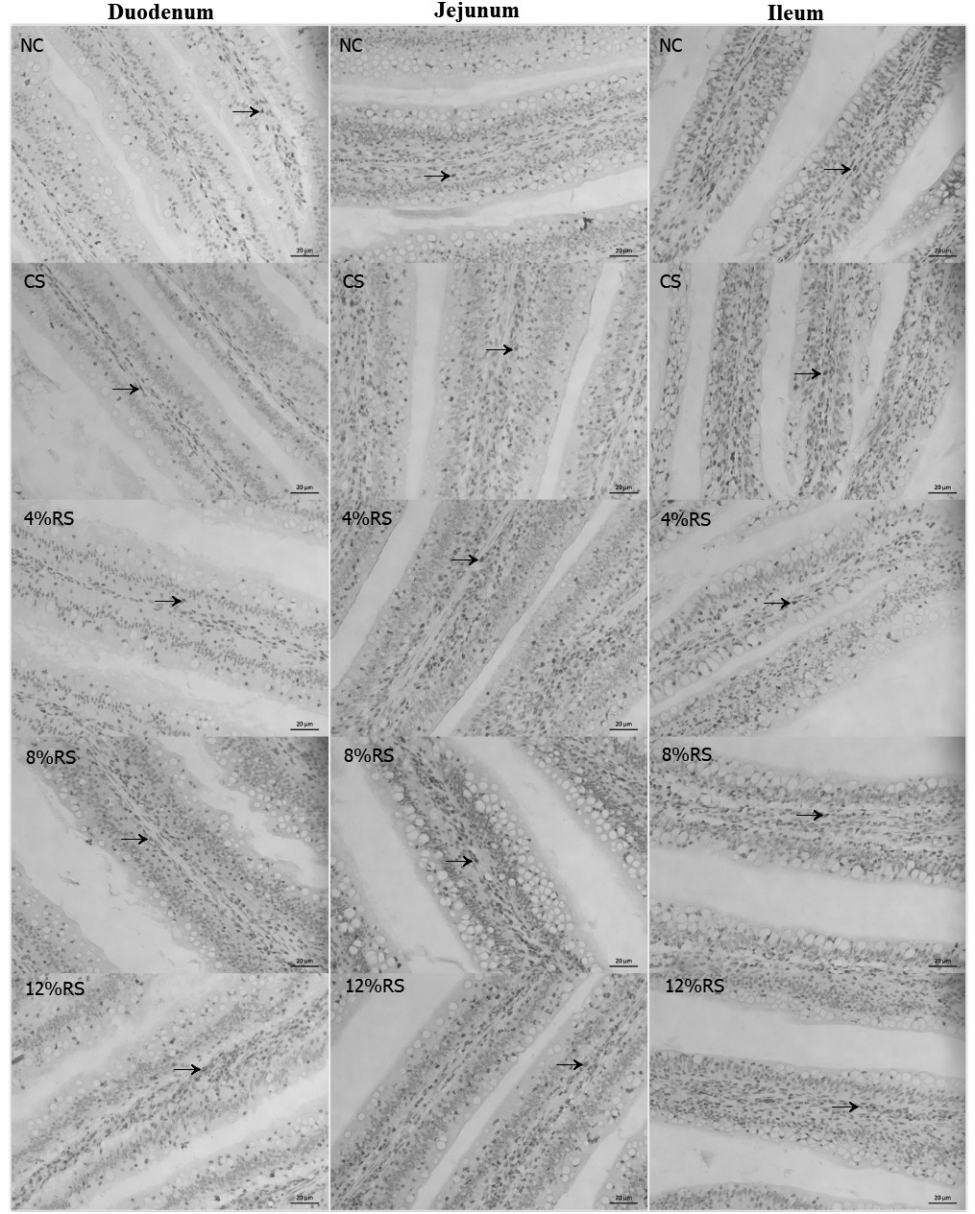
The PCNA immunohistochemical assessment of duodenum, jejunum and ileum villus of 21-day-old broilers (Arrows point to PCNA-positive cells).NC, a basic normal corn–soybeandiet; CS, a corn–soybean–based diet supplementation with 20% corn starch (CS); 4% RS, 8% RS, and 12% RS, the corn–soybean–based diets supplementation with 4%, 8%, and 12% corn resistant starch (RS), respectively; PCNA, proliferating cell nuclear antigen. Image, magnification at ×400.

PCNA immunoreactivity was observed in duodenum, jejunum and ileum of broilers (Table 8, Figure 2). The percentage of duodenal PCNA-positive cells was higher in 12% RS group than that of the control (p<0.01). Feeding CS and RS treated diets increased the percentage of duodenal PCNA-positive cells in jejunum, and the birds only fed with 8% RS and 12% RS diets had an increased ileal percentage of PCNA-positive cells, compared to the control (p<0.01). Furthermore, the percentage of PCNA-positive cells in duodenum and ileum were increased linearly by feeding RS treated diets to broilers (p<0.01).

### Mucin glycoproteins and tight junction proteins mRNA expression levels in jejunal mucosa

**Figure 3:**
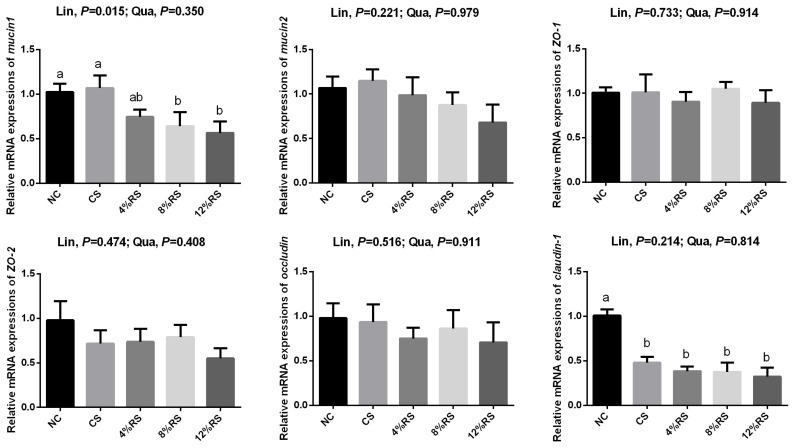
Effects of corn resistant starch on the relative mRNA expressions of *mucin1*, *mucin2*, *claudin-1*, *occludin*, *ZO-1*, and *ZO-2* in jejunal mucosa of 21-day-old broilers. Results are represented as the mean value±standard error of the mean (n = 8). ^a,b^ Means in a row without a common superscript letter significantly differ (p<0.05). NC, a basic normal corn–soybean diet; CS, a corn–soybean–based diet supplementation with 20% corn starch (CS); 4% RS, 8% RS, and 12% RS, the corn–soybean–based diets supplementation with 4%, 8%, and 12% corn resistant starch (RS), respectively; *ZO-1*, zonula occludens-1; *ZO-2*, zonula occludens-2.

The *mucin1* mRNA expressions in 8% RS and 12% RS groups and the *claudin1* mRNA expressions in CS and RS groups were lower than those of NC group (p<0.05, Figure 3). The mRNA expression of *mucin1* was decreased linearly in response to the increased dietary RS level (p<0.05).

### Notch signal pathway mRNA expression levels in jejunum mucosa

**Figure 4:**
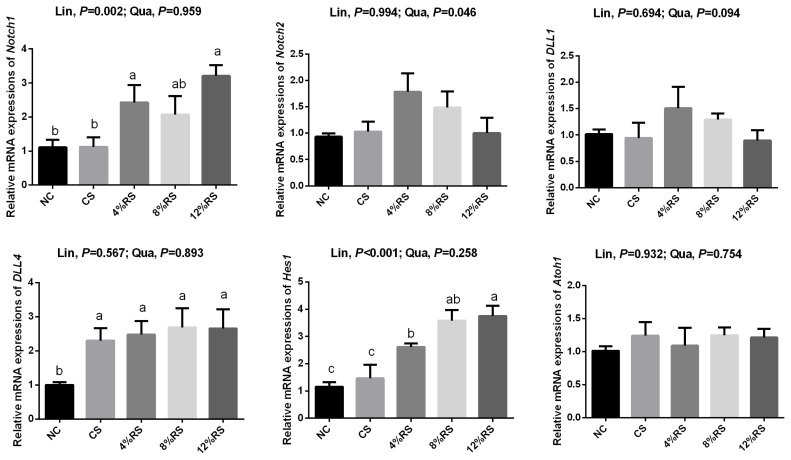
Effects of corn resistant starch on the relative mRNA expressions of *notch1*, *notch2*, *DLL1*, *DLL4*, *Hes1*, and *Atoh1* in jejunal mucosa of 21-day-old broilers. Results are represented as the mean value±standard error of the mean (n = 8). ^a–c^ Means in a row without a common superscript letter significantly differ (p<0.05). NC, a basic normal corn–soybean diet; CS, a corn–soybean–based diet supplementation with 20% corn starch (CS); 4% RS, 8% RS, and 12% RS, the corn–soybean–based diets supplementation with 4%, 8%, and 12% corn resistant starch (RS), respectively. *DLL1*, delta like canonical Notch ligand 1; *DLL4*, delta like canonical Notch ligand 4; *Hes1*, hes family bHLH transcription factor 1; *Atoh1*, atonal homolog 1.

According to Figure 4, the mRNA expression of *Notch1* was increased by feeding 4% RS and 12% RS diets compared to control (p<0.05). Birds from RS groups exhibited higher mRNA expression of delta like canonical Notch ligand 4 (*DLL4*), and the mRNA expression of hes family bHLH transcription factor 1 (*Hes1*) was merely increased in RS treated groups, compared with the NC group (p<0.05). Additionally, feeding increased RS diets linearly up-regulated the mRNA expressions of *Notch1* and *Hes1* (p<0.01).

### Plasma diamine oxidase activity and D-lactic acid concentration

There was no significant difference on the plasma DAO activities and D-lactic acid concentration among NC, CS, and all RS groups (Table 9).

## DISCUSSION

Corn is the most used cereal grain in the diets of intensively reared poultry, and CS contributes about 60% of the apparent metabolisable energy content of poultry feeds [22]. The RS content is always higher in the CS with a higher content of amylose [23]. Due to the indigestibility of RS, animal growth performance is often decreased when feeding corn with high RS ratio, Bergh et al [24] reported that broiler chickens fed with a high amylose diet exhibit a decreased BW, feed intake and an increased F/G ratio. A similar result was obtained from the present study, all CS and RS groups had a lower feed intake and BW gain as well as a higher F/G ratio compared with the NC group. One reason for this may be the supplementation of RS in diets can effectively increase the concentrations of peptide YY and glucagon-like peptide-1 [25,26], these hormones usually can suppress appetite and reduce food intake, further decreasing weight gain [27]. In addition, our previous study also found that RS significantly decreased nutrient digestibility [28], which might also contribute to a lower BW gain.

Intestinal tract is the main site for digestion and absorption of nutrients. In the present study, we found that birds fed CS and RS diets had higher relative lengths of small intestine (duodenum, jejunum, and ileum) than those fed the control diet. This most likely enabled the chickens to absorb nutrients efficiently as the digesta would take longer to pass through the intestine as the absorption area become larger. Meanwhile, it should be noted that intestinal absorptive capacity depends more on overall villus height [29]. Unexpectedly, all RS treatments significantly decreased jejunal VH in broilers. The result was opposite to the finding by Qin et al [30] who found that RS significantly improved the intestinal morphology of ducks. The main reason may be that ducks have stronger intestinal microbial fermentation capacity than chickens and can use more indigestible carbohydrates [31]. A lower villus would decrease the areas of nutrient absorption. Hence, we speculated that RS may decrease the digestion and absorption of nutrients in the jejunum. The jejunal VH/CD was decreased linearly in response to the increase of dietary RS level, which again proved that the higher dietary RS level was, the worse nutrients digestion and absorption ability would be.

The RS is resistant to degradation in the small intestine but metabolized by microbes in the hindgut, where they are fermented into SCFAs, including acetic acid, propionic acid and butyric acid [5]. Among the SCFAs, butyrate has been investigated most extensively. Butyrate is the primary energy source for epithelial cells and functions as a histone deacetylase inhibitor [32], which alters the expression of many genes with diverse functions, some of which include cell proliferation and differentiation [15]. In this study, all RS treatments increased the butyric acid concentration, which may promote epithelial cell proliferation. PCNA plays an essential role in DNA replication, DNA repair and cell cycle control [33,34], and has a broad correlation with mitotic activity, for which it can be used as a marker for cell proliferation in intestinal mucosa. The current result showed that 12% RS treatments increased the percentage of PCNA-positive cells of the villus in duodenum, jejunum and ileum. This suggested that high dietary RS can promote the proliferation of intestinal epithelium cells of broilers. As mentioned above, the intestinal mucosal epithelium consists of four main cell types—absorptive enterocytes, goblet cells, Paneth cells, and enteroendocrine cells [17]. Intestinal goblet cells are responsible for the release of mucins into the intestinal lumen. We wondered if the increased epithelium proliferation would affect the differentiation of various cell types. Therefore, the goblet cells density in the small intestine was observed. The result showed that all RS treatments decreased the duodenal and jejunal goblet cells density. It is intestinal epithelium stem cells residing in the base of crypts that give rise to a transit-amplifying population of cells that undergo rapid proliferation and differentiation into the various intestinal epithelial cell subsets [15]. The decreased goblet cells indicated that RS may suppress the goblet cell differentiation and promote other cells type, which may have a profound impact on the physiological functions of the intestine

Notch signaling pathway plays a central role in cell fate differentiation towards secretory versus non-secretory lineage [15]. Previous studies have found that Notch inhibition led to precocious differentiation of epithelial progenitors into secretory cell types, including large numbers of cells that expressed both Paneth and goblet cell [12]. Conversely, activation of Notch signaling in intestinal epithelium will repress secretory cell differentiation [2]. To determine whether RS affect the development of intestinal epithelium by regulating the Notch pathway, the relative mRNA expressions of *Notch1*, *Notch2*, *DLL1*, *DLL4*, *Hes1*, and *Atoh1* in jejunal mucosa were assessed. We found that the *Notch1*, *DLL4*, and *Hes1* mRNA expressions of jejunal mucosa of the all RS groups were higher than those of the NC group. These results demonstrated that RS activated the Notch pathway, which may be responsible for the reduction of secretory goblet cells.

The mucus layer covers the entire gastrointestinal tract, mainly because of the secretory products of goblet cells [17]. Mucin1 and mucin2 are membrane-bound mucins and gel-forming secretory mucins, respectively. They provide the host's first line of defense against endogenous or exogenous attack and microbial attachment and invasion, but allow nutrients to pass through [17,35]. In current study, dietary RS reduced the density of duodenal goblet cells, which may lead to a decrease in mucin production. Therefore, we detected the relative mRNA expressions of *mucin1* and *mucin2* in jejunal mucosa. Not surprisingly, the relative mRNA expressions of *mucin1* were decreased in 8% RS and 12% RS groups, which may have a negative effect on intestinal barrier function.

The mucosal barrier is mainly divided into mechanical barriers, biological barriers, chemical barriers and immunological barriers [36]. The mechanical barrier plays an important role in absorbing nutrients and resisting invasion of enteric pathogens. While the mechanical barrier is formed by epithelial cells (e.g. absorptive cells and goblet cells) and the junctional complex, consisting of tight junctions (TJ), adherens junctions, gap junctions, and desmosomes [3,37]. Occludin, claudins and zonula occludens (ZO) are the most important components in the regulation of epithelial barrier function among TJ. Therefore, we also examined the relative mRNA expressions of *claudin-1*, *occludin*, *ZO-1*, and *ZO-2* in jejunal mucosa. Similarly, the relative mRNA expression of *claudin-1* and *ZO-1* were also decreased. It's common sense that intestinal permeability is managed by TJ [38], so the decreased TJ might alter intestinal permeability.

Finally, DAO and D-lactic acid in plasma were measured to test intestinal permeability. The DAO is an enzyme that catalyzes the oxidation of diamines. It is normally abundant in intestinal mucosa, but rarely in other tissues. When intestinal mucosa is damaged, DAO activity in the blood increases. Hence, serum DAO activity might be a good marker for intestinal mucosal integrity [39]. In this study, there was no significant difference in the plasma DAO activities among all groups, indicating that although dietary RS inhibited the development of duodenal goblet cell, mucins and TJ, but it was not enough to damage intestinal permeability. In addition, D-lactic acid is the fermentation product of various bacteria in intestinal tract. Like DAO, the concentration of plasma D-lactic acid will increase when the intestinal mucosa is damaged. Hence, D-lactic acid can also be used as a marker for intestinal mucosal integrity. However, there was also no significant difference in the plasma D-lactic acid concentration in all groups. This again demonstrates that mucosal permeability has not been impaired despite the goblet cell development and barrier function were inhibited.

## CONCLUSION

In summary, dietary RS can impair intestinal morphology and barrier functions. The molecular mechanisms of the impaired barrier functions could be due to the increase of intestinal mucosa cell proliferation, which causes an inhibited development of goblet cells by activating notch pathway and results in decreased mucins and TJ mRNA expressions.

## Figures and Tables

**Table 1:** Ingredients and nutrient composition of experimental diets (as fed basis)

Items	Treatments1)				

	NC	CS	4% RS	8% RS	12% RS
Ingredients (%)					
Corn	57.00	36.50	36.50	36.50	36.50
Soybean meal (44% crude protein)	31.50	28.15	28.15	28.15	28.15
Corn gluten meal (63.5% crude protein)	3.40	8.15	8.15	8.15	8.15
Corn starch	-	20.00	13.33	6.67	-
Hi-Maize 260 (60% resistant starch)	-	-	6.67	13.33	20.00
Soybean oil	3.10	2.20	2.20	2.20	2.20
Limestone	1.20	1.20	1.20	1.20	1.20
Dicalcium phosphate	2.00	2.00	2.00	2.00	2.00
L-lysine	0.34	0.34	0.34	0.34	0.34
DL-methionine	0.15	0.15	0.15	0.15	0.15
Salt	0.30	0.30	0.30	0.30	0.30
Zeolite powder	0.01	0.01	0.01	0.01	0.01
Premix2)	1.00	1.00	1.00	1.00	1.00
Calculated nutrient levels					
Metabolizable energy (MJ/kg)	12.52	12.50	-	-	-
Crude protein (%)	21.33	21.00	21.00	21.00	21.00
Calcium (%)	1.00	1.00	1.00	1.00	1.00
Available phosphorus (%)	0.46	0.45	0.45	0.45	0.45
Lysine (%)	1.21	1.12	1.12	1.12	1.12
Methionine (%)	0.50	0.51	0.51	0.51	0.51
Methionine + cysteine (%)	0.86	0.85	0.85	0.85	0.85
Arginine (%)	1.27	1.18	1.18	1.18	1.18
Threonine (%)	0.83	0.81	0.81	0.81	0.81
Analysed nutrient levels					
Crude protein (%)	20.91	20.40	20.61	20.39	20.64
Starch (%)	51.25	51.23	51.34	52.30	52.01
RS (%)	3.03	4.18	7.33	11.02	14.16

1) NC, a basic normal corn–soybean diet; CS, a corn–soybean–based diet supplementation with 20% corn starch (CS); 4% RS, 8% RS, and 12% RS, the corn–soybean–based diets supplementation with 4%, 8%, and 12% corn resistant starch (RS), respectively.

2) Premix provided per kilogram of diet: trans-retinyl acetate, 30 mg; cholecalciferol, 0.075 mg; DL-α-tocopherol acetate, 30 mg; menadione, 1.3 mg; thiamine, 2.2 mg; riboflavin, 8.0 mg; nicotinamide, 40 mg; choline, 400 mg; pantothenic acid (D-Ca pantothenate), 15 mg; pyridoxine HCl, 4mg; biotin, 0.04 mg; folic acid, 1 mg; cobalamin, 0.013 mg; Fe (from ferrous sulphate), 80 mg; Cu (from copper sulphate), 8.0 mg; Mn (from manganese sulphate), 110 mg; Zn (from zinc sulphate), 60 mg; I (from calcium iodate), 1.1 mg; Se (from sodium selenite), 0.3 mg.

**Table 2:** Primer sequences for real-time quantitative PCR analysis

Genes	GenBank numbers	Primer sequences (5′→3′)	Product sizes (bp)
*Mucin-1*	XM_015279046.2	Forward: ACGCCTTCTTCAGCAGCAACTC Reverse: AGCAGCAGATGTGAGCAGTGATG	183
*Mucin-2*	NM_001318434.1	Forward: ACTGGACTTCACGGACACCT Reverse: CCCCCTCTACCATCATCAAA	121
*Claudin-1*	NM_001013611.2	Forward: GACCAGGTGAAGAAGATGCGGATG Reverse: CGAGCCACTCTGTTGCCATACC	107
*occludin*	XM_025144248.1	Forward: CTGCTCTGCCTCATCTGCTTCTTC Reverse: CCATCCGCCACGTTCTTCACC	143
*ZO-1*	XM_015278981.2	Forward: GCCAGCCATCATTCTGACTCCAC Reverse: GTACTGAAGGAGCAGGAGGAGGAG	172
*ZO-2*	NM_204918.1	Forward: GAGAGCACAACCGAAGCAGAGG Reverse: TAGTCCTGTCCATAGCCACCATCC	157
*Notch1*	NM_001030295.1	Forward: GACAGCATCGCCGCCTTCAC Reverse: CGTCCAGGTTGATCTCGCAGTTG	187
*Notch2*	NM_001252033.1	Forward: CCAAAGCGAACCCTTGTTACC Reverse: CACACCCAAGAGAAGAATCAAGAG	94
*DLL1*	NM_204973.2	Forward: CTGGTCCTCATGCTGCTGCTG Reverse: CACCGATGACGCTGATGGAGATG	166
*DLL4*	XM_421132.6	Forward: GCTACTGTCCTTCTGGCTTCATGG Reverse: CATCGTCTCCAAGTCCTGCTGTG	200
*Hes1*	XM_015291592.2	Forward: GGACGCGCTGAAGAAGGATAGTTC Reverse: GCAGGTGCTTGACGGTCATCTC	80
*Atoh1*	XM_004941130.3	Forward: GGCTGAACCACGCCTTCGAC Reverse: GCGTCTCGTACTTGGAGAGCTTC	81

*ZO-1*, zonula occludens-1; *ZO-2*, zonula occludens-2; *DLL1*, delta like canonical Notch ligand 1; *DLL4*, delta like canonical Notch ligand 4; *Hes1*, hes family bHLH transcription factor 1; *Atoh1*, atonal homolog 1.

**Table 3:** Effect of corn resistant starch on the growth performance of broilers from 0 to 21 days

Items	Treatments1)					SEM	p-value		
	
	NC	CS	4% RS	8% RS	12% RS		ANOVA	Linear2)	Quadratic2)
Feed intake (g/bird)	966.76^a^	726.68^b^	733.07^b^	761.29^b^	762.75^b^	15.96	<0.001	0.003	0.788
BW gain (g/bird)	678.26^a^	482.72^b^	480.06^b^	497.63^b^	471.25^b^	13.53	<0.001	0.594	0.103
Feed/gain (g/g)	1.43^c^	1.50^b^	1.53^b^	1.53^b^	1.62^a^	0.01	<0.001	<0.001	0.005

Results are represented as the mean value±SEM (n = 8).

SEM, standard error of the mean; ANOVA, analysis of variance; BW, body weight.

1) NC, a basic normal corn–soybean diet; CS, a corn–soybean–based diet supplementation with 20% corn starch (CS); 4% RS, 8% RS, and 12% RS, the corn–soybean–based diets supplementation with 4%, 8%, and 12% corn resistant starch (RS), respectively.

2) Orthogonal polynomial contrast was used to determine linear and quadratic effects of increasing concentrations of resistant starch in CS and RS diets.

a–c Means in a row without a common superscript letter significantly differ (p<0.05).

**Table 4:** Effects of corn resistant starch on the relative length and weight of small intestine of 21-day-old broilers

Items	Treatments1)					SEM	p-value		
	
	NC	CS	4% RS	8% RS	12% RS		ANOVA	Linear2)	Quadratic2)
Duodenum (cm/kg BW)	35.68^d^	41.67^c^	43.91^bc^	45.79^ab^	47.57^a^	0.66	<0.001	<0.001	0.822
Jejunum (cm/kg BW)	78.15^b^	108.85^a^	110.29^a^	113.55^a^	121.95^a^	3.19	<0.001	0.126	0.592
Ileum (cm/kg BW)	73.81^b^	108.85^a^	110.38^a^	112.73^a^	121.96^a^	3.40	<0.001	0.155	0.579
Duodenum (g/kg BW)	6.88^b^	6.87^b^	6.51^b^	8.10^a^	8.50^a^	0.16	<0.001	<0.001	0.196
Jejunum (g/kg BW)	16.23^b^	17.94^ab^	18.23^a^	19.83^a^	19.04^a^	0.75	0.002	0.108	0.293
Ileum (g/kg BW)	12.23	12.45	12.97	13.03	14.55	1.33	0.178	0.068	0.511

Results are represented as the mean value±SEM (n = 8).

SEM, standard error of the mean; ANOVA, analysis of variance; BW, body weight.

1) NC, a basic normal corn–soybean diet; CS, a corn–soybean–based diet supplementation with 20% corn starch (CS); 4% RS, 8% RS, and 12% RS, the corn–soybean–based diets supplementation with 4%, 8%, and 12% corn resistant starch (RS), respectively.

2) Orthogonal polynomial contrast was used to determine linear and quadratic effects of increasing concentrations of resistant starch in CS and RS diets.

a–d Means in a row without a common superscript letter significantly differ (p<0.05).

**Table 5:** Effect of corn resistant starch on the intestinal morphology of 21-day-old broilers

Items	Treatments1)					SEM	p-value		
	
	NC	CS	4% RS	8% RS	12% RS		ANOVA	Linear2)	Quadratic2)
Duodenum									
VH (μm)	839.60	840.12	800.98	836.23	813.06	14.97	0.893	0.709	0.625
CD (μm)	107.50^a^	107.08^a^	87.89^b^	89.58^b^	82.75^b^	2.56	<0.001	<0.001	0.118
VH/CD (μm/μm)	8.48	8.28	8.93	9.62	9.51	0.19	0.067	0.006	0.235
Jejunum									
VH (μm)	705.68^a^	663.84^c^	681.17^b^	670.78^bc^	679.31^bc^	3.25	<0.001	0.166	0.443
CD (μm)	102.73^b^	101.15^b^	102.97^b^	102.74^b^	105.26^a^	0.35	<0.001	<0.001	0.594
VH/CD (μm/μm)	6.87^a^	6.56^bc^	6.62^b^	6.53^cd^	6.45^d^	0.03	<0.001	0.065	0.038
Ileum									
VH (μm)	444.13^ab^	334.46^c^	378.85^bc^	435.16^ab^	478.90^a^	14.94	0.012	0.001	0.662
CD (μm)	98.07^a^	62.67^c^	62.90^c^	75.23^bc^	87.63^ab^	3.27	<0.001	<0.001	0.195
VH/CD (μm/μm)	5.12	5.68	6.39	6.05	5.82	0.21	0.425	0.750	0.544

Results are represented as the mean value±SEM (n = 8).

SEM, standard error of the mean; ANOVA, analysis of variance; VH, villus height; CD, crypt depth; VH/CD, the ratio of villus height to crypt depth.

1) NC, a basic normal corn–soybean diet; CS, a corn–soybean–based diet supplementation with 20% corn starch (CS); 4% RS, 8% RS, and 12% RS, the corn–soybean–based diets supplementation with 4%, 8%, and 12% corn resistant starch (RS), respectively.

2) Orthogonal polynomial contrast was used to determine linear and quadratic effects of increasing concentrations of resistant starch in CS and RS diets.

a–d Means in a row without a common superscript letter significantly differ (p<0.05).

**Table 6:** Effect of corn resistant starch on the intestinal goblet cell density of 21-day-old broilers

Items	Treatments1)					SEM	p-value		
	
	NC	CS	4% RS	8% RS	12% RS		ANOVA	Linear2)	Quadratic2)
Duodenum (n/per 100 μm villus)	6.71^a^	6.38^ab^	5.79^bc^	5.67^bc^	5.31^d^	0.15	0.009	0.166	0.053
Jejunum (n/per 100 μm villus)	7.50	6.75	6.71	6.64	7.08	0.11	0.061	0.480	0.371
Ileum (n/per 100 μm villus)	7.07	7.30	7.00	7.43	7.44	0.08	0.290	0.442	0.024

Results are represented as the mean value±SEM (n = 8).

SEM, standard error of the mean; ANOVA, analysis of variance.

1) NC, a basic normal corn–soybean diet; CS, a corn–soybean–based diet supplementation with 20% corn starch (CS); 4% RS, 8% RS, and 12% RS, the corn–soybean–based diets supplementation with 4%, 8%, and 12% corn resistant starch (RS), respectively.

2) Orthogonal polynomial contrast was used to determine linear and quadratic effects of increasing concentrations of resistant starch in CS and RS diets.

a–d Means in a row without a common superscript letter significantly differ (p<0.05).

**Table 7:** Effect of corn resistant starch on the concentrations of short chain fatty acids in cecal digesta of 21-day-old broilers

Items	Treatments1)					SEM	p-value		
	
	NC	CS	4% RS	8% RS	12% RS		AVONA	Linear2)	Quadratic2)
Acetate (μM/g)	30.55^b^	49.37^a^	48.67^a^	49.61^a^	52.83^a^	2.46	0.026	0.640	0.720
Propionate (μM/g)	1.84^c^	2.14^bc^	3.09abc	3.40^ab^	3.85^a^	0.21	0.011	0.009	0.536
Butyrate (μM/g)	2.62^b^	5.42^a^	5.64^a^	6.19^a^	6.40^a^	0.29	<0.001	0.170	0.978

Results are represented as the mean value±SEM (n = 8).

SEM, standard error of the mean; ANOVA, analysis of variance.

1) NC, a basic normal corn–soybean diet; CS, a corn–soybean–based diet supplementation with 20% corn starch (CS); 4% RS, 8% RS, and 12% RS, the corn–soybean–based diets supplementation with 4%, 8%, and 12% corn resistant starch (RS), respectively.

2) Orthogonal polynomial contrast was used to determine linear and quadratic effects of increasing concentrations of resistant starch in CS and RS diets.

a–c Means in a row without a common superscript letter significantly differ (p<0.05).

**Table 8:** Effect of corn resistant starch on the percentage of PCNA-positive cells in the intestinal villus of 21-day-old broilers

Items	Treatments1)					SEM	p-value		
	
	NC	CS	4% RS	8% RS	12% RS		AVONA	Linear2)	Quadratic2)
Duodenum (percentage of PCNA-positive cells, %)	16.97^bc^	13.12^d^	15.63^cd^	20.03^ab^	21.24^a^	0.63	<0.001	<0.001	0.562
Jejunum (percentage of PCNA-positive cells, %)	18.78^b^	21.54^a^	23.37^a^	21.92^a^	23.52^a^	0.40	<0.001	0.247	0.707
Ileum (percentage of PCNA-positive cells, %)	25.81^b^	26.85^b^	28.13^b^	34.09^a^	33.42^a^	0.85	0.001	0.002	0.610

Results are represented as the mean value±SEM (n = 8).

PCNA, proliferating cell nuclear antigen; SEM, standard error of the mean; ANOVA, analysis of variance.

1) NC, a basic normal corn–soybean diet; CS, a corn–soybean–based diet supplementation with 20% corn starch (CS); 4% RS, 8% RS, and 12% RS, the corn–soybean–based diets supplementation with 4%, 8%, and 12% corn resistant starch (RS), respectively.

2) Orthogonal polynomial contrast was used to determine linear and quadratic effects of increasing concentrations of resistant starch in CS and RS diets.

a–d Means in a row without a common superscript letter significantly differ (p<0.05).

**Table 9:** Effects of corn resistant starch on the plasma diamine oxidase activity and D-lactic acid concentration of 21-day-old broilers

Items	Treatments1)					SEM	p-value		
	
	NC	CS	4% RS	8% RS	12% RS		AVONA	Linear2)	Quadratic2)
DAO (U/L)	21.71	19.61	22.29	21.01	19.70	0.74	0.754	0.671	0.730
D-lactic acid (nmol/mL)	10.11	11.33	11.4	10.18	9.93	0.72	0.952	0.806	0.514

Results are represented as the mean value±SEM (n = 8).

SEM, standard error of the mean; ANOVA, analysis of variance; DAO, diamine oxidase.

1) NC, a basic normal corn–soybean diet; CS, a corn–soybean–based diet supplementation with 20% corn starch (CS); 4% RS, 8% RS, and 12% RS, the corn–soybean–based diets supplementation with 4%, 8%, and 12% corn resistant starch (RS), respectively.

2) Orthogonal polynomial contrast was used to determine linear and quadratic effects of increasing concentrations of resistant starch in CS and RS diets.
